# Supplementary material for: Splice-Junction-Based Mapping of Alternative Isoforms in the Human Proteome
Source: Cell Rep. Author manuscript; Available in PMC 2020 Jan 15. (PMC6961840; doi:10.1016/j.celrep.2019.11.026)
Supplement: 2 [file NIHMS1546469-supplement-2.zip › DF1/PXD006675/LeftAtrium/LeftAtrium_26_RAB1A_GAHGIIVVYDVTDQEFADSLGIPFLETSAK.pdf]

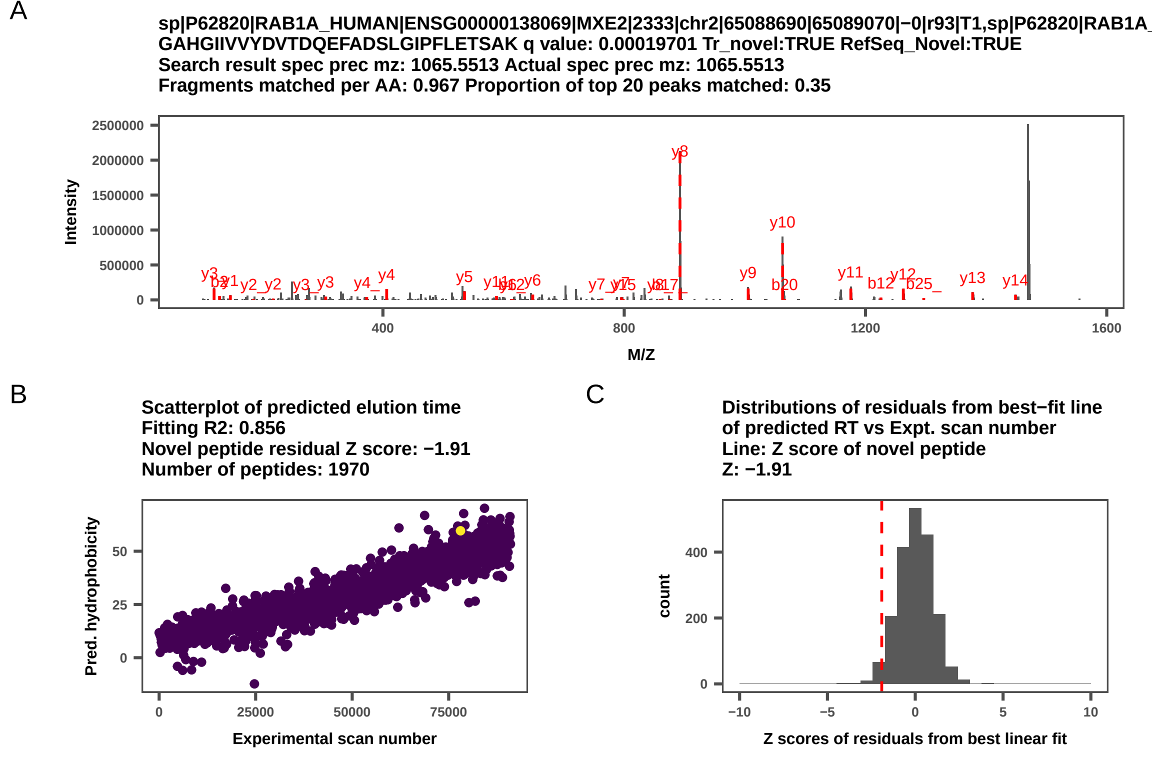

A
sp|P62820|RAB1A_HUMAN|ENSG00000138069|MXE2|2333|chr2|65088690|65089070|−0|r93|T1,sp|P62820|RAB1A_
GAHGIIVVYDVTDQEFADSLGIPFLETSAK q value: 0.00019701 Tr_novel:TRUE RefSeq_Novel:TRUE
Search result spec prec mz: 1065.5513 Actual spec prec mz: 1065.5513
Fragments matched per AA: 0.967 Proportion of top 20 peaks matched: 0.35
Intensity
M/Z
B
Scatterplot of predicted elution time
Fitting R2: 0.856
Novel peptide residual Z score: −1.91
Number of peptides: 1970
Pred. hydrophobicity
Experimental scan number
C
Distributions of residuals from best−fit line
of predicted RT vs Expt. scan number
Line: Z score of novel peptide
Z: −1.91
count
Z scores of residuals from best linear fit
